# A Synergistic Anti-Cancer Effect of Troglitazone and Lovastatin in a Human Anaplastic Thyroid Cancer Cell Line and in a Mouse Xenograft Model

**DOI:** 10.3390/ijms19071834

**Published:** 2018-06-22

**Authors:** Wen-Bin Zhong, Yuan-Chin Tsai, Li-Han Chin, Jen-Ho Tseng, Li-Wen Tang, Steve Horng, Yu-Ching Fan, Sung-Po Hsu

**Affiliations:** 1 Department of Physiology, School of Medicine, College of Medicine, Taipei Medical University, Taipei 110, Taiwan; wbzhong@tmu.edu.tw; 2 Graduate Institute of Medical Sciences, College of Medicine, Taipei Medical University, Taipei 110, Taiwan; d119096017@tmu.edu.tw (L.-H.C.); m120105020@tmu.edu.tw (L.-W.T.); 3 Graduate Institute of Cancer Biology and Drug Discovery, College of Medical Science and Technology, Taipei Medical University, Taipei 110, Taiwan; yuanchin@tmu.edu.tw (Y.-C.T.); steve4211@tmu.edu.tw (S.H.); ssfan1616@gmail.com (Y.-C.F.); 4 Department of Neurosurgery, Taipei City Hospital, Renai Branch, Taipei 106, Taiwan; DAL87@tpech.gov.tw

**Keywords:** anaplastic thyroid cancer, HMGCR, lovastatin, PPARγ, p21^cip^, p27^cip^, RB, troglitazone

## Abstract

Anaplastic thyroid cancer (ATC) is a malignant subtype of thyroid cancers and its mechanism of development remains inconclusive. Importantly, there is no effective strategy for treatment since ATC is not responsive to conventional therapies, including radioactive iodine therapy and thyroid-stimulating hormone suppression. Here, we report that a combinational approach consisting of drugs designed for targeting lipid metabolism, lovastatin (an inhibitor of 3-hydroxy-3-methylglutaryl coenzyme A reductase, HMGCR) and troglitazone (an agonist of peroxisome proliferator-activated receptor gamma, PPARγ), exhibits anti-proliferation in cell culture systems and leads to tumor regression in a mouse xenograft model. The composition contains a sub-lethal concentration of both drugs and exhibits low toxicity to certain types of normal cells. Our results support a hypothesis that the inhibitory effect of the combination is partly through a cell cycle arrest at G0/G1 phase, as evidenced by the induction of cyclin-dependent kinase inhibitors, p21^cip^ and p27^kip^, and the reduction of hyperphosphorylated retinoblastoma protein (pp-Rb)-E2F1 signaling. Therefore, targeting two pathways involved in lipid metabolism may provide a new direction for treating ATC.

## 1. Introduction

Anaplastic thyroid cancer (ATC) is a devastating subtype of thyroid cancers [1]. Patients have short survival time after diagnosis [2], and the leading cause of death is usually associated with airway hindrance or metastasis-related complications. Surgery remains an important approach; however, the involvement of other crucial structures (e.g., esophagus) complicates the procedure and presents difficulty in a complete resection [3,4]. Other therapeutic approaches including radiotherapy and chemotherapy encounter challenges like collateral toxicity and drug resistance. In general, conventional therapies neither improve the life quality of patients nor provide significant benefits for treatment outcomes [5]. Therefore, novel strategies for ATC treatment are still in demand.

Lovastatin is approved for treating cholesterol-related diseases by inhibiting the rate-determining enzyme, 3-hydroxy-3-methylglutaryl coenzyme A reductase (HMGCR), in the mevalonate (MEV) pathway [6]. Beneficial functions of lovastatin have been shown in treating vascular-related diseases [7] and glomerulonephritis [8] by reducing plasma cholesterol. In addition, an increasing body of research has indicated that lovastatin reduces cancer cell growth via inducing cell cycle arrest and apoptosis [9,10]. Recently, studies in clinical settings showed that statin drugs abrogate cancer progression, supporting their pleiotropic effects and the idea of targeting the MEV pathway in cancer treatment [11,12].

The peroxisome proliferator-activated receptor gamma (PPARγ) is a member of the steroid hormone receptor superfamily. As a ligand-activated nuclear transcriptional factor, PPARγ regulates the homeostasis of lipid and glucose. Thiazolidinediones (TZDs) are a class of agonists of PPARγ and are mainly used in treating type 2 diabetes by attenuating insulin resistance [13,14]. In addition to re-sensitizing insulin function, TZDs have been shown to inhibit proliferation and facilitate apoptosis and differentiation in several types of cancers [15]. Emerging evidence has also shown that the combination of TZDs with other types of drugs may be a new direction for anti-tumor activities [16].

Previously, we demonstrated that treatment with lovastatin alone inhibited cell proliferation and migration, and enhanced apoptosis and re-differentiation in an ARO (UCLA RO-81-A-1) cell line [17,18,19,20]. Here, we investigated the anti-cancer effects in ATC cells in response to the combination of lovastatin and troglitazone. We found that the combination of a sub-lethal concentration of both lovastatin and troglitazone has a tumor-specific anti-proliferation effect in vitro and in vivo. We hypothesize that the inhibitory effect of this combination treatment is partly through cell cycle arrest at the G0/G1 phase. Since one hurdle in treating cancer is its heterogeneity, our results provide a promising strategy for ATC treatment by targeting two independent metabolic pathways (HMGCR and PPARγ).

## 2. Results

### 2.1. Combination of Troglitazone and Lovastatin Shows Tumor-Specific Anti-Proliferation Effect

**Figure 1:**
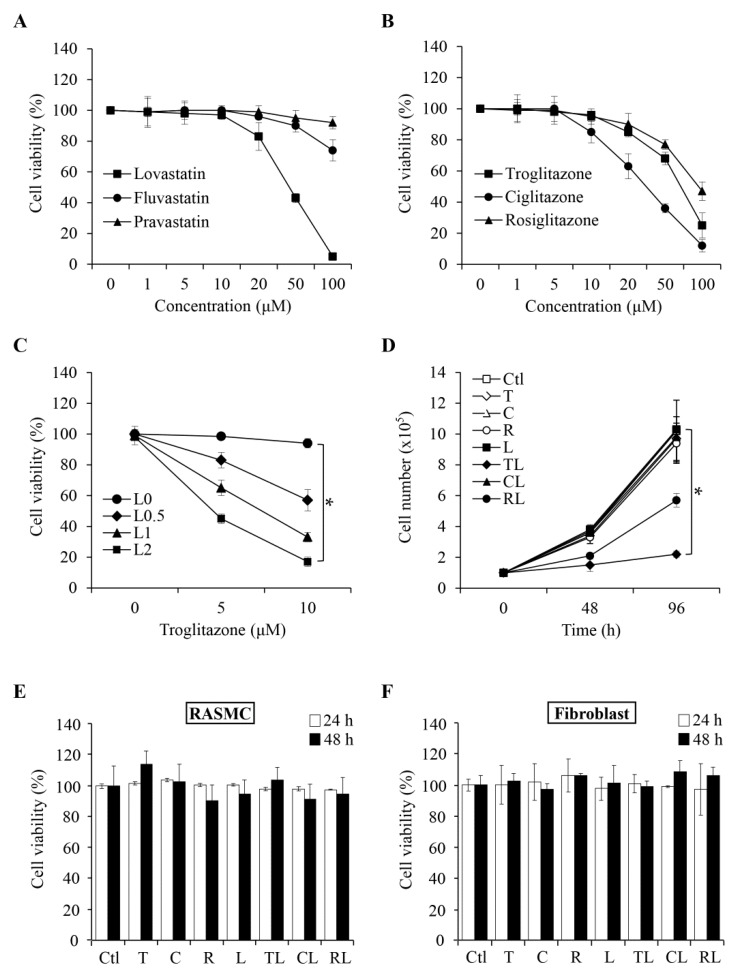
A synergistic effect in the anti-proliferation activity of troglitazone and lovastatin. (**A**) Concentration responses of three statin derivatives in suppressing proliferation in SW1736 cells. (**B**) Concentration responses of three thiazolidinediones (TZDs) derivatives in SW1736 cells. (**C**) Monitoring the anti-proliferation effect of troglitazone in combination with lovastatin. Cells were treated with troglitazone (5 and 10 μM) in combination with lovastatin (labeled L, 0.5, 1, and 2 μM). (**D**) Monitoring the proliferation rates in SW1736 cells treated with different combinations of indicated drugs. Concentrations used were defined previously. Single agents (T, C, R, and L): 10 μM except L (2 μM). Combination (TL, CL, and RL): TZDs (10 μM) plus L (2 μM). Cells were treated for 48 h and 96 h, and then analyzed by trypan blue staining. (**E**,**F**) Lack of anti-proliferation effect in normal RASMC (**E**) and human gingival fibroblast cells (**F**). Cells were treated with indicated drugs as used in (**D**) for 24 and 48 h. Proliferation was analyzed by 3-(4,5-Dimethylthiazol-2-yl)-2,5-diphenyltetrazolium bromide (MTT) assay. Values represent the means ± S.E.M. (*n* = 3). * *p* < 0.05. C: ciglitazone; CL: combination of ciglitazone and lovastatin; Ctl: control; L: lovastatin; R: rosiglitazone; RL: combination of rosiglitazone and lovastatin; T: troglitazone; TL: combination of troglitazone and lovastatin.

To examine the anti-proliferation effects of statins (lovastatin, fluvastatin, pravastatin) and TZDs (roglitazone, ciglitazone, and rosiglitazone drugs) in ATC SW1736, cells were treated with a range of concentrations for 48 h. As shown in Figure 1A, lovastatin significantly reduced cell viability in a concentration-dependent manner compared to either fluvastatin or pravastatin. For TZD class, ciglitazone exhibited a stronger inhibitory effect than troglitazone and rosiglitazone (Figure 1B). In examining the effects of combination treatments, we found that there was a synergism between lovastatin and troglitzone (Figure 1C). We therefore analyzed different statins as single agents and TZDs in combination with lovastatin. As shown in Figure 1D, the combination of troglitazone and lovastatin displayed the strongest anti-proliferation effect. We also examined whether the combination treatment led to similar anti-proliferation effects in normal cells. Using human umbilical vein endothelial cells (HUVEC) as one of the in vitro models, we found that lovastatin at a concentration of 2 μM alone can inhibit HUVEC proliferation (Supplementary Figure S1). In addition, three TZD drugs in combination with lovastatin also caused a 30% to 50% decrease in cell proliferation as compared to the control group at 48 h (Supplementary Figure S1). We also examined the same panel of drug combinations in two additional normal cells, rat aortic smooth muscle cell (RASMC) and human gingival fibroblast. In contrast to the inhibitory effects observed in HUVEC and SW1736, we found that both normal cells were not affected by the combination of troglitzone and lovastatin (Figure 1E,F). Based on these results, in addition to ATC SW1736, the combination could also exhibit tissue-specific anti-proliferation effects.

### 2.2. Inhibitory Effect of Combination Treatment Is Reversible

**Figure 2:**
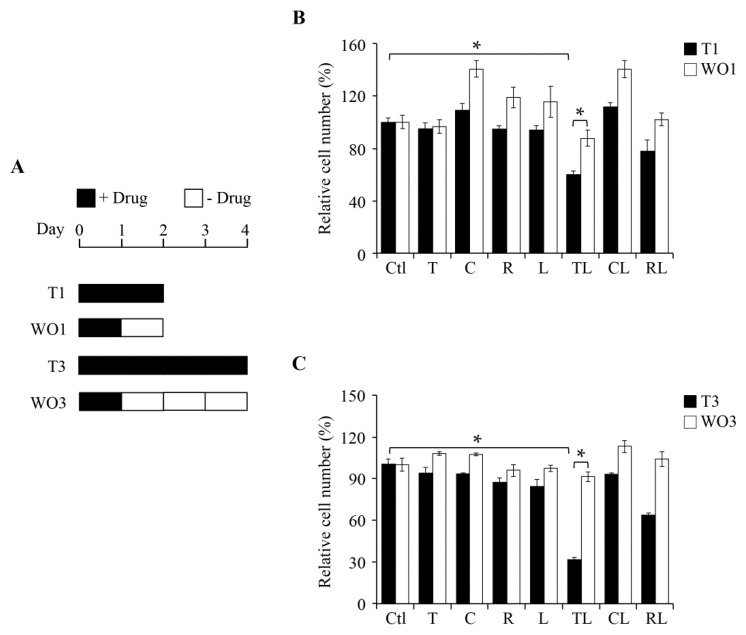
Reversible feature of the anti-proliferation effect following drug-removal. (**A**) The experimental procedures for treatment and drug-removal. Cells were treated with the indicated drugs for 1 day, followed by washout with fresh medium and incubation for an additional 1 (WO1) or 3 (WO3) day(s). (**B**,**C**) Proliferation assay in cells with or without drug-removal for 1 day (**B**) or 3 days (**C**). The drug concentration used is the same as that in Figure 1D. The relative cell number was estimated by MTT assay. Values represent the means ± S.E.M. (*n* = 3). * *p* < 0.05. C: ciglitazone; CL: combination of ciglitazone and lovastatin; Ctl: control; L: lovastatin; R: rosiglitazone; RL: combination of rosiglitazone and lovastatin; T: troglitazone; TL: combination of troglitazone and lovastatin.

We further tested whether this anti-proliferation effect is reversible. The SW1736 cells were treated with the drugs indicated for 1 day followed by drug-removal for either 1 day (WO1) or 3 days (WO3) (Figure 2A). As illustrated in Figure 2B, we found that 1-day drug-removal rescued around 20% of SW1736 (TL). Furthermore, 3-day drug-removal can increase survival close to 70% (TL, Figure 2C). Together, these findings suggest that the inhibitory effect on ATC SW1736 cells following the co-treatment of troglitazone and lovastatin is reversible.

### 2.3. Combination of Sub-Lethal Concentrations of Lovastatin and Troglitazone Leads to Cell Cycle Arrest at the G0/G1 Phase

**Figure 3:**
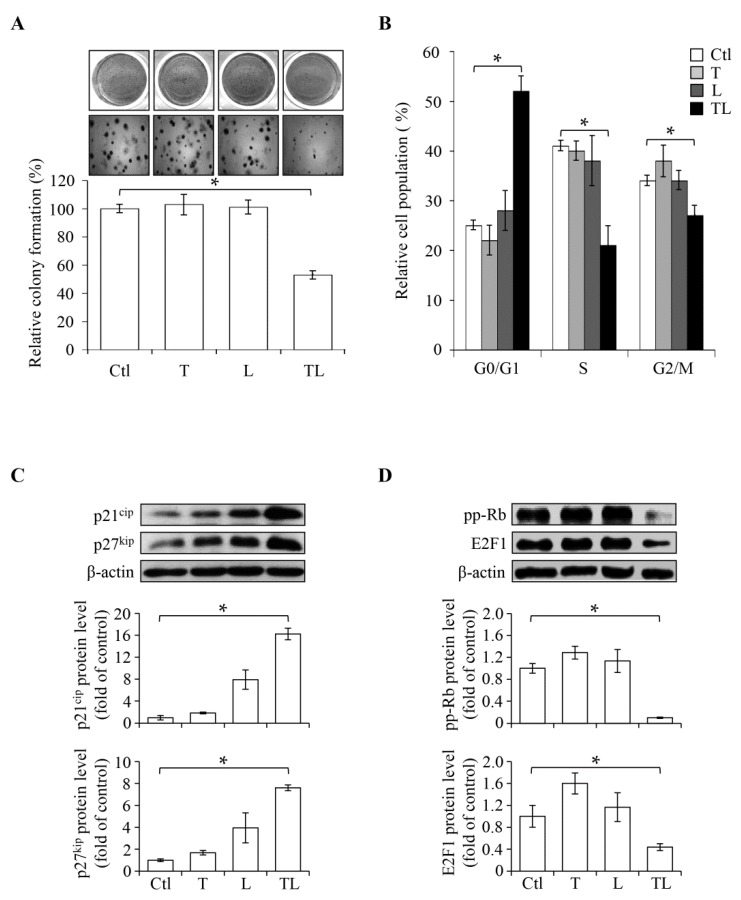
Combination treatment of troglitazone and lovastatin inhibits cell cycle progression. (**A**) Combination treatment of troglitazone (T) and lovastatin (L) restrained the anchorage-independent growth of SW1736 cells in semi-solid agar. Cells were treated with the indicated drugs for 14 days and then the relative cell colony formation was assayed. Cell colonies were fixed and then stained by crystal violet (magnification ×40). (**B**) The combination treatment increased the relative ratio of cell populations accumulating at the G0/G1 phase. Cells were treated with the indicated drugs for 24 h and then the DNA content was analyzed by flow-cytometry. (**C**,**D**) Combination treatment induced the protein expression levels of p21^cip^ and p27^kip^ (**C**) and inhibited the hyperphosphorylated (pp)-Rb and E2F1 (**D**). Upper panels: immunoblotting images. Lower panels: quantification of immunoblotting. Cells were treated with the indicated drugs for 24 h and analyzed by immunoblotting. Values represent the means ± S.E.M. (*n* = 3). * *p* < 0.05. Ctl: control; L: lovastatin; (2 μM) T: troglitazone (10 μM); TL: combination of troglitazone and lovastatin.

SW1736 is a malignant ATC cell line. We examined its anchorage-independent growth, which reflects the metastatic capacity, by performing a colony formation assay. As shown in Figure 3A, the colonies were reduced by co-treatment with previously defined sub-lethal concentrations (2 μM lovastatin and 10 μM troglitazone), confirming again the synergistic effect on anti-proliferation. We further analyzed the effect of combination treatment (T + L, Figure 3B) on cell cycle progression; the ratio of cell populations at each cell cycle phase was analyzed. As demonstrated in Figure 3B, the cell population at the G0/G1 phase was increased from 30% to 50%, and that at the S phase was reduced from 40% to 20%, suggesting a regulatory mechanism at the G0/G1 phase arrest. Earlier studies reported that the levels of cell cycle inhibitors, p21^cip^ [21,22] and p27^kip^ [23], were reduced and the Rb/E2F1 activity was increased in ATC cells [24]. Therefore, we further examined the cellular responses following combination treatment by monitoring these key regulators at the G1/S checkpoint. Consistent with the observed G0/G1 arrest, co-treatment showed increased expression of p21^cip^ and p27^kip^ (Figure 3C) and decreased expression of G1/S promoters, including hyperphosphorylated Rb (pp-RB) and E2F1 (Figure 3D). We also found that the upregulation of p21^cip^ and p27^kip^ is dependent on de novo translation and transcription since both actinomycin D (AcD) and cycloheximide (CHX) remarkably suppressed the induction effects by the combination (Supplementary Figure S2). We also confirmed that SW1736 challenged with the combination treatment, followed by the 1-day drug-removal procedure, rescued the E2F1 level (Supplementary Figure S3), thus supporting the idea that the inhibitory effect is reversible. Taken together, our results suggest that co-treatment with troglitazone and lovastatin induces cell cycle arrest at the G0/G1 phase in ATC cells.

### 2.4. Combination of Troglitazone and Lovastatin Leads to Tumor Regression in a Mouse Xenograft Model

**Figure 4:**
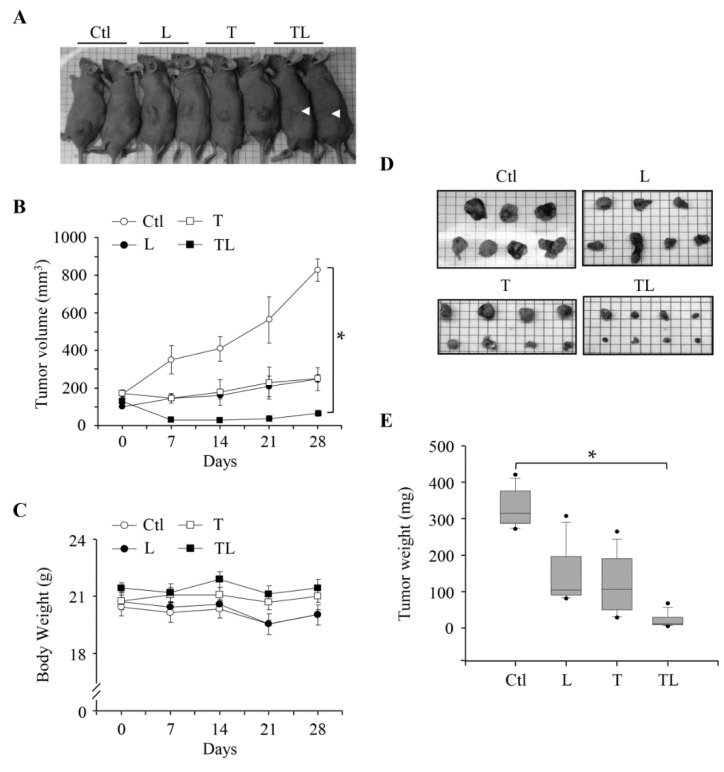
Combined administration of troglitazone and lovastatin restrained tumor progression in a mouse xenograft model. Human ATC SW1736 cells were subcutaneously inoculated in the left-lower dorsum of Bagg’s albino (BALB)/c nude mice and the cells were allowed to grow up and form a solid tumor. After tumors reached up to a size of ~200 mm^3^, phosphate-buffered saline (PBS) or drugs were administered daily via intraperitoneal injection, five times a week for 4 weeks. (**A**) Represented mice bearing tumor xenografts. (**B**) Monitoring the tumor volume following drug administration. (**C**) The body weight of mice among each group was not significantly changed during treatment. (**D**) The solid tumors in each group at the end of treatment. (**E**) Measurement of the tumor weight in mice bearing tumor xenografts following drug treatments. Values represent the means ± S.E.M. (Ctl: *n* = 7; T: *n* = 8; L: *n* = 7; TL: *n* = 8). Black dot: single data point. Arrowhead: reduction in tumor size. * *p* < 0.05. Ctl: control; L: lovastatin (2 mg/kg/day); T: troglitazone (5 mg/kg/day); TL: combination of troglitazone and lovastatin.

Since the combination of troglitazone and lovastatin at their sub-lethal concentrations can exhibit cell-specific anti-proliferation activities, we next examined whether this combination could also suppress tumor growth in vivo. First, the SW1736 cell line was subcutaneously inoculated in nude mice, allowing a tumor to reach a certain range of size (~200 mm^3^). Then, the tumor-bearing mice were randomly divided into four groups: the control (injection with phosphate-buffered saline (PBS)), troglitazone (labeled T), lovastatin (labeled L), and combination (TL) treatments. Compared to single-agent treatment (T and L), which prevented the tumor growth and maintained the initial size, the combination efficiently suppressed the progression and caused tumor regression (Figure 4A,B). Notably, body weights of mice at each group did not alter during the 28-day experimental period, suggesting no toxic side effect on normal tissues (Figure 4C). Furthermore, the combination approach markedly suppressed tumor growth in the xenograft model by monitoring tumor volume and weight (Figure 4D,E). These findings demonstrated that the combination of both drugs exhibits an effective anti-tumor effect in vivo.

## 3. Discussion

**Figure 5:**
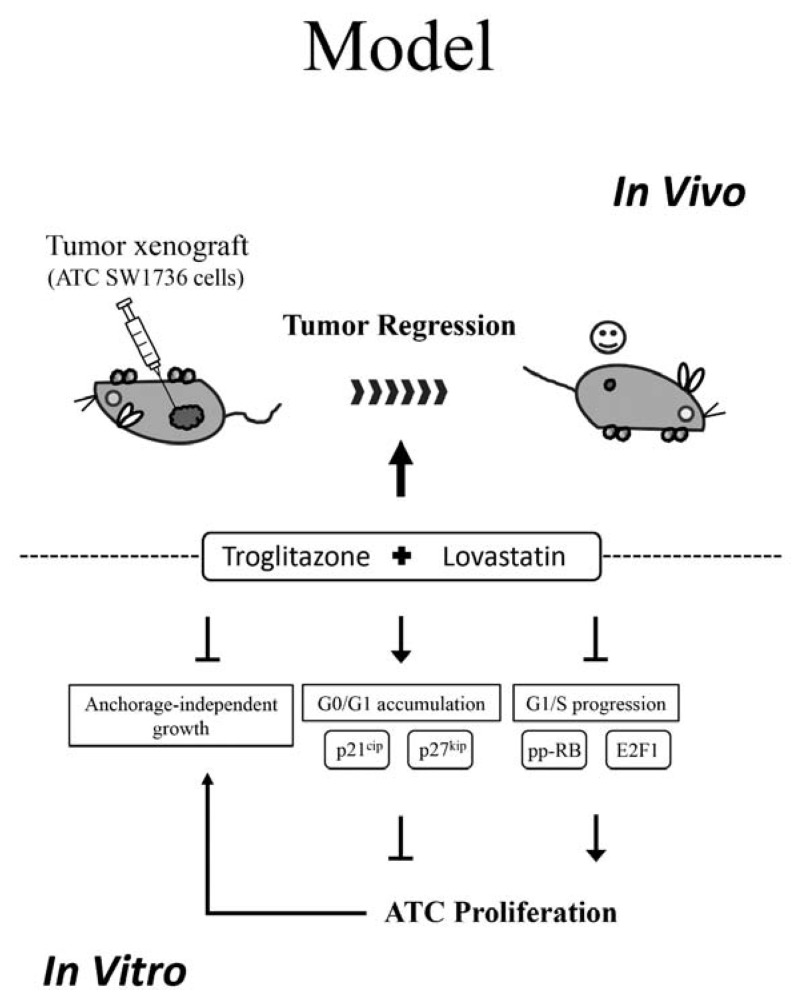
A working model for the anti-tumor effects by combination of troglitazone and lovastatin. Combination treatment inhibits tumor growth and induces cell cycle arrest at the G0/G1 phase. The underlying mechanism is partly achieved through the upregulation of p21^cip^ and p27^kip^, and partly through the reduction of pp-RB and E2F1 expression. Arrow: promotion; T-bar: inhibition.

A great deal of advances in genomic analysis and immunotherapy have been made in past decades. However, therapies against ATC have not been improved [25]. One approach to accelerate the discovery is by drug repositioning [26]. In this study, we explored the therapeutic effects by a combination of troglitazone and lovastatin. Both drugs have biochemically defined targets, lovastatin for HMGCR and troglitazone for PPARγ, and exhibit anti-tumor effects when combined in a sub-lethal concentration (Figure 1 and Figure 4). We also showed that the combination can suppress signaling responsible for the G1/S progression (pp-RB and E2F1) and induce the expression of p21^cip^ and p27^kip^ (Figure 3). Based on these findings, we propose that the mechanism underlying the combination of troglitazone and lovastatin-induced anti-tumor effect is possibly due to the disruption of G1/S progression and cell cycle arrest at the G0/G1 phase (Figure 5).

Although statins are originally designed for treating cardiovascular disease, several lines of evidence have shown their anti-cancer effects. However, there are differential activities among statins regarding anti-proliferation in different cancer cell lines. For instance, it has been shown that fluvastatin, lovastatin, and simvastatin inhibit several breast cancer cell lines, while pravastatin has no effect [27]. In leukemia cells, cerivastatin was shown to have better inhibitory effects than fluvastatin or lovastatin [28]. Our results in an ATC cell line, SW1736, showed that lovastatin has a stronger anti-proliferation effect than fluvastatin and pravastatin, suggesting that lovastatin has better specificity than the other two in treating ATC (Figure 1A). This could be an advantage in future drug development for targeting ATC.

TZDs are specific pharmaceutical ligands for PPARγ, and have been shown to be useful in treating thyroid cancers, through the re-activation of radioiodine-uptake (ClinicalTrials.gov Identifier: NCT00098852) and the promotion of re-differentiation (ClinicalTrials.gov Identifier: NCT01655719) of thyroid cancers [16]. The tumor suppression activity of PPARγ has been intensively studied in numerous human malignancies (including breast, brain, colon, lung, pancreas, prostate and tongue malignancies) as well as observed in preclinical models [29,30]. It is worth noting that the anti-proliferation effects among the TZDs as single agents (ciglitazone > troglitazone > rosiglitazone) are different from their combination with lovastatin (i.e., troglitazone > rosiglitazone > ciglitazone) (Figure 1B,D). It is not yet clear whether the combination of lovastatin and troglitazone triggers the strongest PPARγ activities compared to other TZDs or other signaling beyond the function of PPARγ in ATC cells. In fact, it has been reported that TZDs also exert anti-cancer effects through a PPARγ-independent mechanism by triggering intracellular calcium flux, inhibition of translation, and cell cycle arrest [31]. The potential “off-target” effects of TZDs or statins might account for the PPARγ-independent functions. Therefore, lovastatin could modulate the intracellular signaling and optimize the role of troglitazone in perturbing proliferation via both the PPARγ-dependent function and a PPARγ-independent mechanism, leading to markedly efficient anti-cancer effects. Further investigation is required to address these issues.

One potential advantage of the combination therapy consisting of lovastatin and troglitazone, other than the markedly efficient anti-cancer effect to ATC, is lower toxicity to normal tissues (Figure 1A,B). In fact, we showed that this combination did not affect two normal tissues, RASMC and human gingival fibroblast (Figure 1E,F). However, the lovastatin and troglitazone combination can lead to decreased proliferation in HUVEC under the same experimental setting (Supplementary Figure S1). One explanation for this tissue-specific toxicity is the different distribution of PPARγ. An earlier study demonstrated that PPARγ is overexpressed in tumor-associated/or proliferating endothelial tissues, and TZDs selectively suppress the proliferation of endothelial cells compared to other types of cell lines [32]. Accordingly, TZDs can prevent primary tumor growth and metastasis possibly through the inhibition of angiogenesis [32]. Since HUVEC belong to one type of endothelial cell, the reduced proliferation following the combination treatment could be similarly due to the PPARγ expression. Indeed, we observed the expression of PPARγ only in ATC SW1763 and HUVEC, but not in RASMC (Supplementary Figure S4). This hypothesis also led us to revisit our result observed in the mouse xenograft model, in that the inhibition of tumor-associated angiogenesis is likely attributable to the observed tumor-regression effect in response to the lovastatin and troglitazone combination (Figure 4). The abundance of PPARγ could be one factor accounting for the tumor- and endothelium-specific anti-proliferation effects. In addition, our earlier studies also showed that lovastatin as a single agent induced apoptosis and re-differentiation in another ATC cell line [17,18,19,20]. Therefore, multiple mechanisms including cell cycle arrest, anti-angiogenesis, and apoptosis may contribute to the anti-cancer effect induced by the lovastatin and troglitazone combination in vivo.

It has been documented that lovastatin can inhibit HMGCR and TZD acts as an agonist for PPARγ. However, it has been reported that both drugs could inhibit Rho kinase. Previously, we demonstrated that lovastatin can inhibit human anaplastic thyroid cancer cell proliferation by reducing Rho geranylgeranylation [18]. Moreover, peroxisome proliferator-activated receptor gamma ligands have been shown to inhibit the Rho/Rho kinase pathway [33]. These findings led us to propose that Rho kinase might be the common target of these two drugs in mediating the cell proliferation inhibition. To prove this possibility, further investigation needs to be done.

The present study demonstrating the synergistic anti-tumor effects of troglitazone and lovastatin against ATC is consistent with an earlier report by Yao et al. [34]. Compared to no effects on some normal tissue (RASMC and human gingiva fibroblast), the combination is partially tumor-specific. In addition, our earlier studies also showed that the combination may prevent epithelial-to-mesenchymal transition and hinder ATC cell migration via inhibition of the expression of cysteine-rich protein 61 [35,36], and the drugs in the combination were shown to have beneficial functions in treating multidrug-resistant cancers [37,38]. Consistent with all these findings, our results support that the combination of troglitazone and lovastatin is a promising approach for treating ATC.

## 4. Materials and Methods

### 4.1. Chemicals, Reagents, and Antibodies

Lovastatin was obtained from Standard Chemical and Pharmacy (Taipei, Taiwan). Fluvastatin was obtained from Novartis (Basel, Switzerland). Pravastatin was a gift from Daiichi Sankyo (Tokyo, Japan). Actinomycin D, ciglitazone, cycloheximide, GW9662, T0070907, and rosiglitazone were purchased from Cayman Chemical (Ann Arbor, MI, USA). Troglitazone was purchased from Tocris Bioscience (Avonmouth, Bristol, UK). Bovine serum albumin (BSA) and 3-(4,5-Dimethylthiazol-2-yl)-2,5-diphenyltetrazolium bromide (MTT) were purchased from Sigma-Aldrich (St. Louis, MO, USA). Unless otherwise indicated, all other chemicals were also purchased from Sigma-Aldrich. DMEM, M199, and RPMI culture media, as well as fetal bovine serum (FBS), kanamycin sulfate, penicillin-streptomycin, and trypsin-EDTA were purchased from Thermo Fisher Scientific (Waltham, MA, USA). Endothelial cell growth supplement (ECGS) was purchased from EMD Millipore (Billerica, MA, USA). Acrylamide/Bis solution and Bradford dye were purchased from Bio-Rad (Hercules, VA, USA). Chemiluminescence reagent plus was purchased from PerkinElmer Life Sciences (Boston, MA, USA). Antibody against E2F1 was obtained from GeneTex (Hsinchu, Taiwan). Antibodies against β-actin were purchased from Jackson Immuno Research Laboratories (West Grove, PA, USA). Antibodies against p21^cip^ and p27^kip^ were purchased from BD Transduction Laboratories (San Diego, CA, USA). Antibody against pp-Rb (S807/811) was purchased from Cell Signaling Technology (Danvers, MA, USA).

### 4.2. Cell Culture

Human ATC SW1736 (a gift from Dr. N. E. Heldin, University of Uppsala, Uppsala, Sweden) cells were grown in RPMI medium containing 10% FBS and 1% penicillin-streptomycin. Human gingival fibroblasts were grown in DMEM containing 10% FBS and 1% penicillin-streptomycin. HUVEC were grown in M199 medium containing 10% FBS, 1% kanamycin, 10 mM HEPES, 5 units/L sodium heparin, and 30 mg/L ECGS. RASMC were grown in DMEM containing 10% FBS and 1% penicillin-streptomycin. All of these cells were incubated at 37 °C in a humidified culture incubator containing 5% CO_2_.

### 4.3. MTT Assay

SW1736 cells (1 × 10^6^ cells/mL) were seeded in 24-well plates for 24 h followed by incubation with the indicated drug compounds at various concentrations for an additional 48 or 96 h. At the end of incubation, a 500 μL volume of MTT reagent (1 mg/mL) was added into each culture well and incubated for 20 min. The medium was then removed, and the intracellular purple formazan dissolved in DMSO was measured at 570 nm in a microtiter plate reader (BioTek Instruments, Winooski, VT, USA). Absorbance at 430 nm served as a reference wavelength.

### 4.4. Soft Agar Colony Formation Assay

SW1736 cells (2 × 10^4^/well) were mixed with 0.36% agarose (BD Difco Agar Noble; Sparks, MD, USA) and plated on the top of 0.72% agarose gel in RPMI 1640 medium containing 10% FBS and 1% penicillin-streptomycin in 6-well plates. Cells were incubated with the indicated drug compounds for 14 days. The cover medium was refreshed every 2 days. Colonies were stained by 0.25% (*w*/*v*) crystal violet. Dark blue cell colonies were counted and presented as an index of anchorage-independent growth.

### 4.5. Flow Cytometry Analysis

The population of SW1736 cells at each phase of the cell cycle was measured by the discriminative DNA content stained with propidium iodide (PI). Cells were fixed with chilled 70% ethanol and kept in a −20 °C container. After 12 h, cells were washed with cold PBS, and then the RNA were removed by the incubation of the cells with 2 mg/mL RNase A at 37 °C for 30 min. To stain cellular DNA, cells were incubated with PI (50 μL/mL solution) at room temperature for 1 h in the dark. DNA content was recorded by a FACSCalibur flow cytometer (Becton Dickinson, San Jose, CA, USA) and then analyzed by CellQuest software v3.3 (Becton Dickinson, Franklin Lakes, NJ, USA).

### 4.6. Immunoblotting Analysis

Total cell lysates were extracted by RIPA lysis buffer (Merck Millipore, Darmstadt, Germany), and then dissolved in 7–12% gels of sodium-dodecyl-sulfate-polyacrylamide-gel-electrophoresis (SDS-PAGE). The proteins were then transferred onto Amersham Hybond-P polyvinylidene difluoride (PVDF) membranes (GE Healthcare Life Science, Piscataway, NJ, USA). The indicated proteins were hybridized with primary antibodies in 3% BSA solution, washed with tris-buffered saline containing 0.1% tween 20, and then probed with horseradish-peroxidase (HRP)-labeled secondary antibodies. The emitted signal of target proteins was developed by chemiluminescence reagent plus and identified by a BioSpectrum Imaging System (UVP, LLC, Upland, CA, USA).

### 4.7. Animal Experiments: Human Tumor Xenograft Models

Animal work was performed in accordance with a protocol approved by the Taipei Medical University Animal Care and Use Committee (approval no: LAC-97-0042, 17 June 2008). The treatment of ATC SW1736-derived xenografts in vivo was performed according to the method described by Wang et al. [39]. For the tumorigenicity assay, 10-week-old BALB/c nude mice (National Laboratory Animal Center, Taipei, Taiwan) were subcutaneously injected with 10^7^ SW1736 cells into the left-lower dorsum. After the tumor size reached a volume of 100–200 mm^3^, mice were administered with different agents five times a week for 28 days: phosphate-buffered saline, lovastatin (2 mg/kg/day), troglitazone (5 mg/kg/day), and lovastatin (2 mg/kg/day) plus troglitazone (5 mg/kg/day). The tumor size was measured weekly with calipers, and the tumor volume was calculated using the following formula: tumor volume = (1/2) × L × W^2^, where L is the length and W the width.

### 4.8. Statistical Analysis

All data were expressed as the mean ± standard error of the mean (SEM). Experimental data were analyzed using one-way analysis of variance (ANOVA) for multiple comparisons or unpaired Student’s *t*-test for comparison between two groups. At a *p*-value < 0.05, significance was statistically accepted. 

## 5. Conclusions

Statins have been widely used for their beneficial activities in cardiovascular disease. TZDs, synthesized PPARγ agonists, are used in controlling blood sugar in patients with diabetes. We demonstrated the synergistic anti-cancer effects of both drugs by using lovastatin and troglitazone in ATC cells. The combinational treatment caused tumor-specific anti-proliferation and cell cycle arrest at the G0/G1 phase. The concept of statin-TZD combination may be a novel and a safer approach compared to its individual ingredient. It is possible to be combined with other modalities for cancer treatment.

## Supplementary Materials

Supplementary materials can be found at http://www.mdpi.com/1422-0067/19/7/1834/s1.
