# Supplementary material for: A Synergistic Anti-Cancer Effect of Troglitazone and Lovastatin in a Human Anaplastic Thyroid Cancer Cell Line and in a Mouse Xenograft Model
Source: Int J Mol Sci. 2018 Jun 22;19(7):1834. doi: 10.3390/ijms19071834 (PMC6073567; doi:10.3390/ijms19071834)
Supplement: Supplementary file 1 [file ijms-19-01834-s001.pdf]

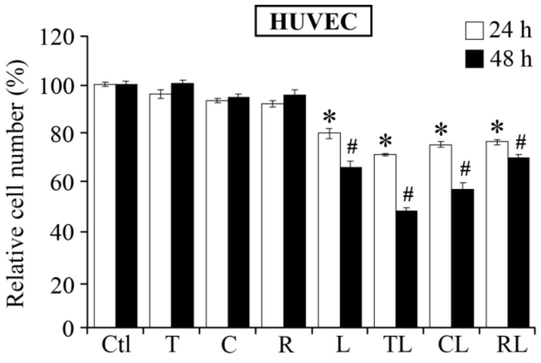

Supplementary Figure S1. Anti-proliferation effect in normal HUVEC. Cells were treated with indicated drugs as used in Figure 1D for 24 h and 48 h. Proliferation was analyzed by MTT assay. Values represent the means ± S.E.M. (n = 3). *P < 0.05, different from control group (24 h). # P < 0.05, different from control group (48 h) C: ciglitazone; CL: combination of ciglitazone and lovastatin; Ctl: control; L: lovastatin; R: rosiglitazone; RL: combination of rosiglitazone and lovastatin; T: troglitazone; TL: combination of troglitazone and lovastatin.

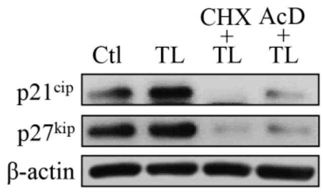

Supplementary Figure S2. Involvement of transcriptional and translational modulation. The protein synthesis inhibitor cycloheximide (CHX) and RNA transcription inhibitor actinomycin D (AcD) abolished the expression of $p21^{cip}$ and $p27^{kip}$ protein. Cells were treated with TL for 12 h followed by addition of AcD (2 μg/ml) or CHX (5 μM) for an additional 12 h. Individual protein expression was measured by immunoblotting assay. Ctl: control; TL: combination of troglitazone (T) and lovastatin (L).

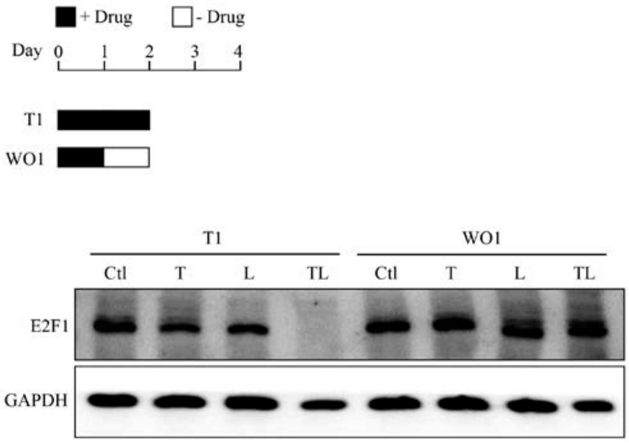

Supplementary Figure S3. Comparison of E2F1 expression in SW1736 cell with drug treatment (T1) and that followed by drug-removal procedure for one day (WO1). The experimental procedures were the same as in Figure 2. Combination treatment (TL) inhibited the E2F1 while one-day washout completely recovered the expression. Ctl: control; L: lovastatin; T: troglitazone; TL: combination of troglitazone (T) and lovastatin (L).

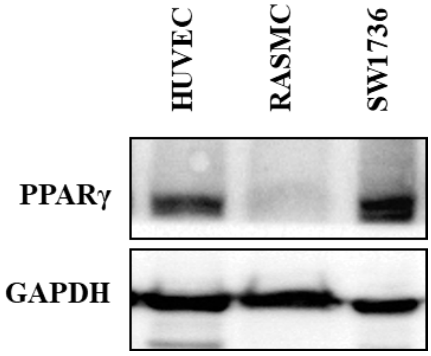

Supplementary Figure S4. Expression of PPARγ in different types of cells. The protein lysates were analyzed by immunoblotting assay using antibody against PPARγ. Antibody against GAPDH was used as loading control. HUVEC: human umbilical vein endothelial cell. RASMC: rat aortic smooth muscle cell. SW1736: human anaplastic thyroid cancer cell.
